# Towards Effective Targeted Alpha Therapy for Neuroendocrine Tumours: A Review

**DOI:** 10.3390/ph17030334

**Published:** 2024-03-04

**Authors:** Paul M. D. Gape, Michael K. Schultz, Graeme J. Stasiuk, Samantha Y. A. Terry

**Affiliations:** 1 School of Biomedical Engineering & Imaging Sciences, King’s College London, London SE1 7EP, UK; graeme.stasiuk@kcl.ac.uk (G.J.S.); samantha.terry@kcl.ac.uk (S.Y.A.T.); 2 Departments of Radiology, Radiation Oncology, Free Radical and Radiation Biology Program, University of Iowa, Iowa City, IA 52242, USA; mschultz@perspectivetherapeutics.com; 3 Perspective Therapeutics, Coralville, IA 52241, USA

**Keywords:** molecular radiotherapy, targeted radionuclide therapy, targeted alpha therapy, peptide receptor radionuclide therapy, neuroendocrine tumours

## Abstract

This review article explores the evolving landscape of Molecular Radiotherapy (MRT), emphasizing Peptide Receptor Radionuclide Therapy (PRRT) for neuroendocrine tumours (NETs). The primary focus is on the transition from β-emitting radiopharmaceuticals to α-emitting agents in PRRT, offering a critical analysis of the radiobiological basis, clinical applications, and ongoing developments in Targeted Alpha Therapy (TAT). Through an extensive literature review, the article delves into the mechanisms and effectiveness of PRRT in targeting somatostatin subtype 2 receptors, highlighting both its successes and limitations. The discussion extends to the emerging paradigm of TAT, underlining its higher potency and specificity with α-particle emissions, which promise enhanced therapeutic efficacy and reduced toxicity. The review critically evaluates preclinical and clinical data, emphasizing the need for standardised dosimetry and a deeper understanding of the dose-response relationship in TAT. The review concludes by underscoring the significant potential of TAT in treating SSTR2-overexpressing cancers, especially in patients refractory to β-PRRT, while also acknowledging the current challenges and the necessity for further research to optimize treatment protocols.

## 1. Introduction

### 1.1. Molecular Radiotherapy

MRT consists of the administration of a radiopharmaceutical, composed of an unstable radionuclide attached to a targeting ligand via a chelator. The ligand binds cellular sites that are overexpressed in tumour cells, but have low expression in healthy cells, therefore delivering cytotoxic radiation specifically to tumour cells and the associated tumour microenvironment, while sparing healthy tissue. As such, MRT has the potential to simultaneously irradiate all cancer cells within the patient, in contrast to local external beam radiation therapies targeting a single site of disease. The use of MRT for the treatment of neuroendocrine tumours has been employed as an effective therapy for several decades. Until recently, MRT targeted to neuroendocrine tumours has primarily employed beta(β)-particle emitters (e.g., ^177^Lu). However, over the last several years, the use of alpha(α)-particle emitters for this application has emerged as potentially transformative. In this review, the transition from β-emitting radiopharmaceuticals to α-emitting agents in PRRT is presented. A critical analysis of the radiobiological basis, clinical applications, and ongoing developments in Targeted Alpha Therapy (TAT) is presented through an extensive literature review that explores this emerging paradigm of TAT and the promise of enhanced therapeutic efficacy and reduced toxicity. The review critically evaluates preclinical and clinical data, emphasizing the need for standardised dosimetry and a deeper understanding of the dose-response relationship in TAT. The review concludes by underscoring the significant potential of TAT in treating SSTR2-overexpressing cancers, especially in patients refractory to β-PRRT, while also acknowledging the current challenges and the necessity for further research to optimize treatment protocols. A complete review of the biology and pathophysiology of neuroendocrine tumours is beyond the scope of this review, which focuses on the use of MRT for the treatment of this disease. Nonetheless, a brief review of this family of malignancies is included here to provide context for the ensuing discussion.

### 1.2. Neuroendocrine Tumours

Neuroendocrine neoplasms are sub-classified by site of origin and pathology. Well-differentiated neoplasms are often referred to as NETs, and poorly differentiated neoplasms as neuroendocrine carcinomas [1]. NETs are further sub-classified according to their Ki-67 proliferation rate as G1, G2 or G3. G1 represents low-proliferative NETs, associated with good prognosis, and G3 represents high grade NETs, associated with poor prognosis [2]. The most common sites of origin are gastroenteropancreatic (GEP) structures and the lung. Tumours can also vary according to their functional status, with some NETs secreting excess hormones. NETs are considered rare cancers, accounting for roughly 0.5% of cancers, but their prevalence has increased in recent years, although it is generally accepted that the increase in prevalence can be attributed to some degree to improved imaging agents [3]. It is common to see NETs referred to as heterogeneous or diverse. This means they arise from a range of tissues and that patients present with a diverse range of symptoms, requiring a multidisciplinary approach to treatment [4]. In patients with localised disease, the first therapeutic option is surgery with curative intent. However, in the case of non-localised (metastasised disease), surgery is generally not considered feasible. In this patient cohort, systemic treatment is necessary and typically starts with a somatostatin analogue (SSA), for example, octreotide or lanreotide. This treatment is not curative in intent but aims to control symptoms. Further treatment options include systemic chemotherapy, though it has been shown that chemotherapy is of limited benefit [5]. This is the point in a patient’s journey at which peptide receptor radionuclide therapy (PRRT) is typically offered [6].

### 1.3. PRRT for NETs—Targeting the Somatostatin Subtype 2 Receptor

**Figure 1:**
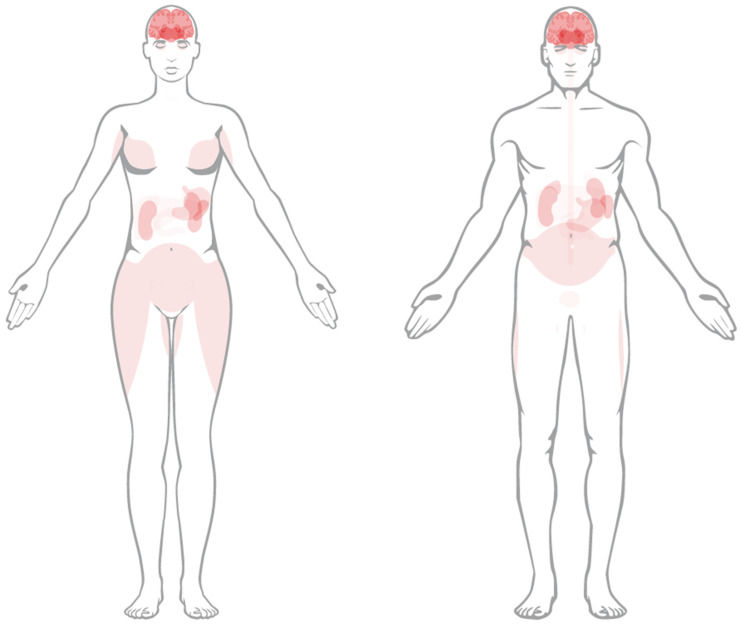
Anatomical depiction of SSTR 2 expression in human tissue in female (**left**) and male (**right**). Image credit: Human Protein Atlas version 23.0 (www.proteinatlas.org/ENSG00000180616-SSTR2/tissue, accessed on 22 February 2024) [9].

PRRT targeting the somatostatin receptor (SSTR) is a specific example of MRT. SSTRs of various subtypes are overexpressed on the cell surface of a range of cancers. Most notably, over 80% of NETs overexpress SSTRs, particularly SSTR subtype 2 (SSTR_2_), making this a suitable target for PRRT [7]. However, as well as being expressed on the surface of neuorendocrine tumour cells, SSTR_2_ is widely expressed in normal tissues, particularly of the endocrine system [8]. Expression of SSTR_2_ in normal tissue is shown anatomically in Figure 1 [9]. Imaging agents targeting SSTR_2_ have shown physiological uptake in the spleen, kidneys, adrenal glands, liver, stomach, and small intestine [10]. While the present discussion is focused on NETs, SSTRs are also expressed in a range of other malignancies, such as lymphoma, several brain tumours, and in breast tumours, which are areas for a future investigation and review [11].

### 1.4. Evolving Standard of Care in PRRT

The most logical choice of targeting ligand may appear to be somatostatin, the native peptide hormone consisting of 14 amino acids. However, the somatostatin peptide-hormone is subject to rapid enzymatic degradation in vivo [12], motivating attempts to develop synthetic somatostatin analogues (SSAs) in order to provide tumour targeting with sufficient stability and affinity.

These earliest iterations of PRRT focused on the development of [^111^In-DTPA-_D_-Phe1]-octreotide ([^111^In]In-pentetreotide), initially for imaging. The utility of the newly developed radiopharmaceutical was initially demonstrated in 1050 patients [13,14]. The uptake demonstrated on imaging subsequently motivated the use of [^111^In]In-pentetreotide for therapy [15]. This demonstrates an early application of the so-called “theragnostic principle”, by which radiolabelled somatostatin analogues can target the same receptor for use in imaging and therapy.

Although the decay of ^111^In results in the release of potentially therapeutic Auger and conversion electrons, the observed efficacy of the ^111^In-labelled agent was modest and several patients developed leukaemia or myelodysplastic syndrome at high activities (>100 GBq) [16]. Given these shortcomings, β-emitters were later favoured due to their higher energy and longer range emissions. Higher energy β-emitters, such as ^90^Y, were considered more promising for the treatment of bulky disease [7], whereas lower energy (thus shorter range) β-emitters, for instance ^177^Lu, result in a lower radiation-absorbed dose to the kidney. ^177^Lu also has the advantage of being directly imageable via the gamma photons in its decay scheme [17].

A novel targeting ligand with higher affinity for SSTR_2_, [Tyr^3^]-octreotide, was developed and combined with the chelator 1,4,7,10-tetra-azacyclododecane-tetra-acetic acid (DOTA), allowing for reliable and stable radiolabelling of ^111^In and ^90^Y ([^90^Y]Y-DOTATOC; as well as ^177^Lu- and ^68^Ga-labelled agents introduced later). The [^90^Y]Y-DOTATOC radiopharmaceutical was shown to be effective in stabilizing disease and slowing the rate of tumour progression, hence becoming the predominant choice of treatment in the early years of PRRT [18,19].

Later iterations of PRRT have utilised a newer somatostatin analogue, DOTA-[Tyr^3^]-octreotate (DOTATATE), due to its higher binding affinity and in vivo uptake in target tissues compared to competitor SSAs [20]. A number of clinical trials have investigated the safety and efficacy of [^177^Lu]Lu-DOTATATE, leading to the international phase three trial, NETTER-1 [21]. This trial established PRRT as standard of care for patients with metastatic SSTR_2_-positive GEP-NETs, demonstrating a significantly higher response rate and extending progression-free survival (PFS) when compared with the control arm (high-dose of long-acting repeatable octreotide administrations). This positive result led to the approval of [^177^Lu]Lu-DOTATATE, under the name Lutathera, in Europe by the European Medicines Agency in late 2017 and in America by the Food and Drug Administration in early 2018 [22]. Notably, while considered safe and effective by these agencies, the objective tumour response rate observed was 18% in the NETTER-1 trial (subsequently revised to 13% in post-trial analysis [1,23]), providing a basis for improvement that is being explored using α-emitting radionuclides. Current clinical practice, as recommended by the joint IAEA, EANM and SNMMI guidance on PRRT, consists of the systemic administration of the radiopharmaceutical over multiple cycles with 6–12 week intervals [7]. Administered activities are generally fixed at 3.7 GBq for [^90^Y]Y-DOTA-TATE/TOC and 5.5–7.4 GBq for [^177^Lu]Lu-DOTATATE and not varied between patients.

Based on the fundamental principle of maximizing the absorbed dose of radiation to tumour tissue, while minimizing radiation exposure to all other normal healthy organs and tissues, the next generation of PRRT may be realised through improvements in targeting ligand, chelator, and/or radionuclide. In the following, the argument for transitioning from β-emitters to α-emitters is considered, and the available literature reviewed critically.

## 2. Targeted Alpha Therapy (TAT)

### 2.1. Radiobiological Basis for TAT

**Figure 2:**
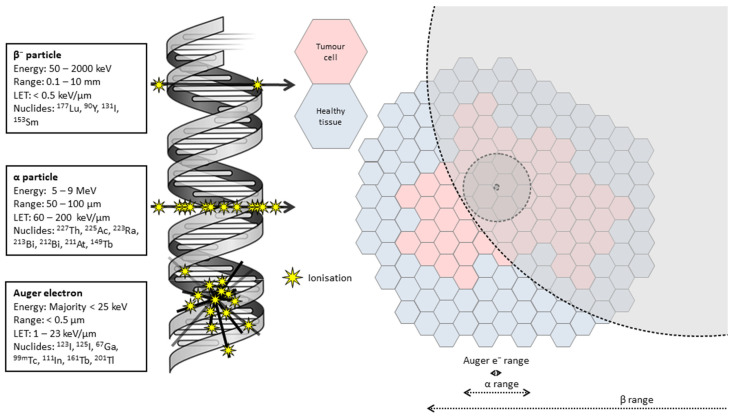
Radiobiological basis for TAT. High LET radiation produces a dense track of ionisations in biological target, producing complex irreparable damage to structures such as DNA. Range of Auger electron, α-particle and β-particle depicted (not to scale) to illustrate potential for sparing of healthy tissue.

TAT is a particularly interesting and promising strategy for cancer treatment given the high potency and specificity associated with α-particle emissions. The α-particle is a helium nucleus consisting of two protons and two neutrons, creating a composite particle with a net positive charge and a mass that is much greater than that of a β^−^-particle (approximately 7000 times greater). The mass and charge of the α-particles make them highly ionizing and limit their range in tissue to 50–100 µm, or approximately 1–3 cell diameters [24]. The energy deposition of the particle along this path, referred to as the linear energy transfer (LET), varies between roughly 60 and 200 keV/µm. For comparison, the LET of a β^−^-particle or a photon ranges from 0.1 to 1.0 keV/µm [25], and the LET of Auger electrons is 4–25 keV/µm [26]. This pattern of energy deposition of α-particles creates a dense track of ionizations along the path of the particle through the biological material in the vicinity of the decay. Where this biological material is the DNA of a tumour cell, these ionizations lead to complex damage such as DNA double-strand breaks, which almost invariably overcome the cell’s repair mechanisms [25]. Conversely, β-emitters are more likely to produce simple, repairable damage, such as well-separated DNA single-strand breaks, due to the sparse nature of their ionization track. Auger electrons produce a dense, irregular pattern of ionisations clustered within several cubic nm from the site of the initial decay. Given the high LET of these low-energy electrons, they also have the potential to produce complex damage that is less reparable than that created by low LET radiation [27]. However, the short range of the Auger electrons means there is a greater requirement for the radionuclide to be transported into the cell and ideally incorporated into the DNA to maximise effectiveness, which may prove difficult in solid tumours [28]. Few α-particles are required to produce a cytotoxic effect, with estimates ranging from 1 to 20 traversals of the nucleus [29,30], resulting in higher potency than lower LET radiations. As demonstrated in Figure 2, the short range of the α-particle and the Auger electron also reduces the irradiation of off-target tissues, potentially reducing the probability of toxicity in normal organs.

Importantly, cell killing with α-emitters is less influenced by oxygen effects than β-emitters, where cytotoxicity is a product of the formation of reactive oxygen species (ROS), which subsequently damage the biological target [31]. The complex nature of the damage caused by individual α-particles also implies that cell survival should not be modulated by dose rate, whereas for low-LET radiation the biological effect of the dose is generally reduced when the dose is given over a longer period of time. This result was shown in vitro as early as 1964 [32]. In the in vivo setting, numerous other factors contribute to the effectiveness of α-emitters in potentiating tumour-specific cell death, including the tumour residence time of the agent and stability of the chelation of the parent and daughter radionuclides in the α-emitter decay chain. Thus, shorter-lived α-emitting radionuclides may provide additional benefit in ensuring a greater percentage of α-particles are localised to tumours within the expected biological residence time of the agent and daughter radionuclides.

The net effect of these differences between radiation qualities is encapsulated by the term relative biological effectiveness (RBE). This parameter is defined as the ratio of absorbed doses of two radiation types required to produce an identical, pre-defined biological effect. For RBE to be a meaningful quantity, the experimental and reference radiations must be defined, dosimetry should be performed, and the biological effect considered should be clearly stated. An RBE value for cell killing by α-particles of between three and five was recommended by a US Department of Energy Panel [33], but the accurate determination of the RBE for clinical and preclinical applications remains an important question. Broadly, the balance between response and toxicity is described by the therapeutic index, calculated as the ratio of the dose at which the treatment can be deemed effective and the dose at which the treatment results in undue toxicity. The therapeutic window may be considered as the difference between these two doses [34]. For the reasons discussed above, it is hoped that TAT will effectively widen the therapeutic window through improved tumour response and reduced off-target toxicity.

### 2.2. Evaluation of Candidate Radionuclides for TAT

There should not be a “one size fits all” approach for TAT. The radionuclide chosen should be matched to the requirements of the indication being treated. One example of this may be to choose an isotope with an appropriate half-life. This depends critically on the biological targeting ligand (sometimes referred to as vector) and the time taken for the radiopharmaceutical to accumulate in target tissues and to be cleared from non-target tissues. As mentioned above, for longer-lived radionuclides, the actual retention time of the agent in the tumour as well as the fate of the decay progeny in the α-emitter decay series must also be considered. The biological clearance of the agent may vary from minutes to days depending on molecular weight (antibodies for example have biological clearance rates that can be measured in days) [35]. Somatostatin analogues are relatively small biomolecules and thus clear quickly from the blood, so may be suited to a shorter half-life isotope [36]. While use of a longer-lived isotope may still be effective, their use is relatively inefficient due to potential washout of the agent from tumours and release of daughter radionuclides from the chelation moiety of the agent.

**Figure 3:**
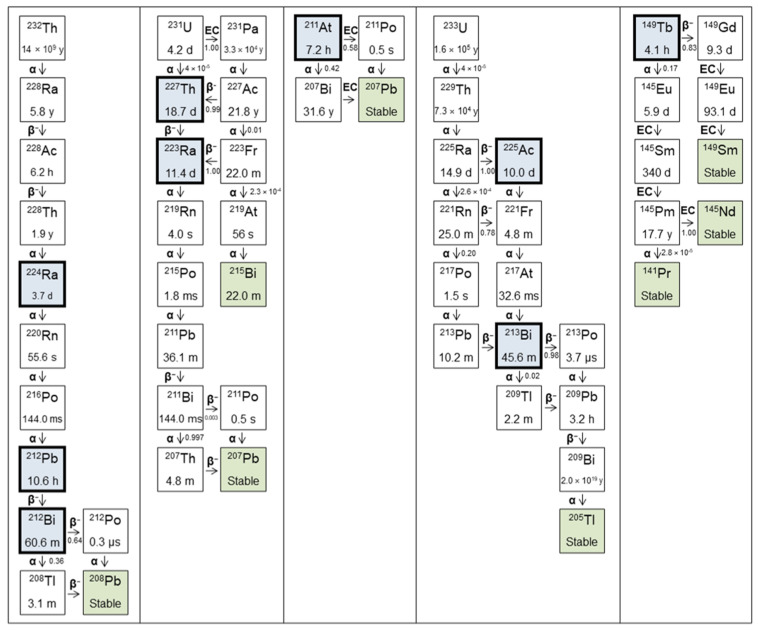
Decay schemes for medically relevant α-emitters. Blue indicates isotopes with potential for clinical translation, green indicates stable isotopes. Decay mode (α, β, electron capture (EC)) indicated, with associated yield where relevant.

The decay schemes for a series of medically relevant α-emitters are shown in Figure 3 and their properties described in Table 1. Also included are so-called ‘in vivo generators’ of α-particles such as ^212^Pb. While technically a β-emitter, the longer half-life of the parent in this case allows for the delivery of the daughter isotope, an α-emitter, to the site of interest in the body [37]. Not all α-emitters are discussed, for example ^226^Th and ^255^Fm. Although these isotopes may have been touted as having potential therapeutic applications, due to difficult production processes, the availability of these isotopes even for research purposes is severely limited [36].

The only α-emitting isotope currently clinically approved in the USA and Europe is ^223^RaCl_2_ and is indicated for castration-resistant prostate cancer and bone metastases (in males), following the results of the ALSYMPCA trial [38]. This represents a simpler scenario, in which the unconjugated radionuclide is injected. ^223^Ra is a calcium mimic and therefore shows accumulation in sites of increased bone turnover, particularly bone metastases. However, this implies that ^223^RaCl_2_ is ineffective against soft tissue metastases. This demonstrates how the efficacy of ^223^Ra could be improved by conjugation with a targeting ligand, though this has been limited due to the relative lack of chelators that demonstrate stability in vivo [39].

Much of the interest in TAT has focused on ^225^Ac. This is principally because of the seven radionuclide daughters in its decay chain (four α and three β^−^), meaning the total decay energy is high relative to other candidate isotopes. ^225^Ac is also readily chelated with DOTA [40], allowing for conjugation with a range of antibodies and small molecules. However, because ^225^Ac is itself an α-emitter, the emission of the α-particle results in the release from chelation of the entire decay series, with each decay. This is due to the recoil energy imparted to the daughter nucleus (^221^Fr), which is more than sufficient to break all chemical bonds with each α-emission by ^225^Ac. This is further exacerbated in that the first daughter in this decay series (i.e., ^221^Fr) has very little affinity for chelation to DOTA-like macrocycles [41]. This leads to a redistribution of the free daughters, with potential to cause significant damage to healthy tissues. For example, ^225^Ac decays to ^213^Bi (through ^221^Fr and ^217^At), and free Bi is known to accumulate in the kidneys [42]. For most studies involving long-lived α-emitters, recoiling daughters pose a serious problem and toxic effects are likely, though the balance between anti-tumour effect and toxicity will vary and must be understood for each isotope, targeting ligand, and indication [43]. This may be less so with the use of relatively short-lived ^212^Pb because the recoil energy of the β-particle emission of ^212^Pb is significantly less than the binding energy of the daughter nucleus to the chelator. Although reports of Bi instability to decoupling has been reported for DOTA and TCMC [44,45], recent reports of a new chelator with improved stability of the chelator ^212^Bi coupling represents an advance that can improve the therapeutic index for targeting ligands conjugated to this new chelator (known as Pb-Specific-Chelator or PSC) [46,47,48,49].

Given the high RBE of the α-particle, knowing the biodistribution of the radiopharmaceutical is of heightened importance to ensure targeted delivery and the minimum off-target exposure. One way to achieve this is through imaging. As shown in Table 1, all the isotopes considered have some potential to be imaged, either directly or via a daughter. However, often this potential is limited by low abundance and complicated decay schemes, as for example with ^223^Ra, where SPECT imaging is feasible but only with long scan times [50]. In a population of patients with metastatic prostate cancer, this may not be tolerable. This limitation can be overcome through imaging with an imaging surrogate isotope. Radionuclides with elementally matched isotopes suitable for imaging offer an advantage here, because the biodistribution of the imaging agent is more likely to be representative of the therapeutic. An example of this principle is seen with ^212^Pb and ^203^Pb, which are suitable for SPECT imaging [47,51]. A recent development that further provides an advantage for the ^212^Pb/^203^Pb-matched pair of isotopes is the demonstration that ^212^Pb SPECT imaging of NET tumours directly is feasible [52]. Quantitative imaging of a tracer amount of the therapeutic radionuclide or a theragnostic pair radionuclide enables treatment planning, as is standard in EBRT, in which target-absorbed doses to tumours and tolerance-absorbed doses to healthy organs are prescribed and the therapeutic activity is calculated to satisfy these constraints [53]. Post-therapy imaging and dosimetry then enable verification of the absorbed doses delivered in MRT.

Aside from the theoretical properties of each candidate radionuclide, translation to the clinic will be limited by availability and supply. The majority of the radionuclides in Table 1 are generator derived. Supply of ^223^Ra via ^227^Ac/^227^Th generators is already established for clinical use. Generators loaded with ^228^Th form the basis of ^224^Ra production and can also be used to supply ^212^Pb and ^212^Bi. While radiolytic damage to the generator matrix material at high activities limits the level of radioactivity that can be loaded to current generators, the availability and half-life of ^228^Th (t_1/2_ = 1.9 years) inventories and the potential for continental distribution of ^224^Ra (t_1/2_ = 3.9 days) enables a system of inventory management and simple wet-chemical purifications that can be readily scaled for commercial radiopharmaceutical production facilities of ^212^Pb radiopharmaceuticals [54]. A number of publications have demonstrated the potential for an emanation-based approach to the production of ^212^Pb via the isolation of gaseous ^220^Rn [55]. However, a production of ^212^Pb using this approach at levels beyond a single clinical dose *per* day has yet to be demonstrated to our knowledge, and current emanation devices would require large inventories of ^228^Th on site at each finished product radiopharmaceutical manufacturing facility. This is an area of intense research as the potential of next generation ^212^Pb-based radiopharmaceuticals is increasingly recognised. To date, the vast majority of ^225^Ac is produced from the decay of ^229^Th, of which only three sources are currently available worldwide. This amounts to approximately 68 GBq *per* year in global production, well below the clinical demand for PRRT [40]. Accelerator and reactor-based approaches are emerging that are showing promise for alleviating this shortfall [56]. For example, the Tri-Lab effort, incorporating US National Laboratories at Oak Ridge, Los Alamos and Brookhaven, will aim to increase supply using accelerator production via ^232^Th(p,x)^225^Ac, with 307 mCi produced in the 2022 financial year and plans to expand production capabilities [57]. Similarly, a partnership between Canadian Nuclear Laboratories (CNL) and German radiopharmaceutical biotech company ITM has been established to increase production by a factor of 30 via irradiation of ^226^Ra targets [58]. Production of ^211^At requires target irradiation by an α-particle beam of energy above 28 MeV. Few accelerator facilities are able to reach this requirement [59]. Clearly, establishment of other production routes to ensure a stable supply of α-emitters for TAT is of critical importance. Continued investment in these technologies for the improved production and purity of ^225^Ac is sought to satisfy the demand for α-emitters for radionuclide therapy.

In the following section, all experience, preclinical and clinical, with TAT of SSTR_2_-overexpressing cancers is critically reviewed.

## 3. Literature Review

### 3.1. Overview

**Figure 4:**
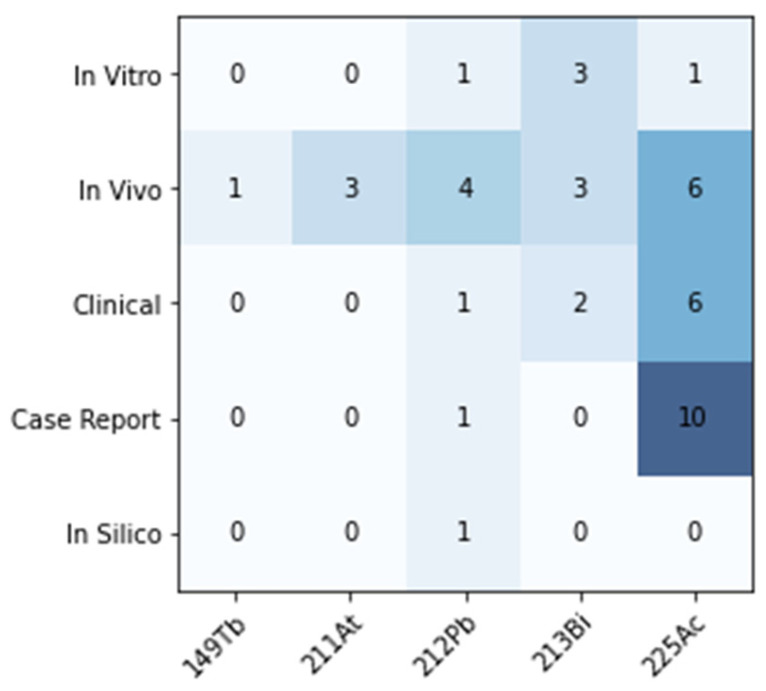
Literature review overview showing the scope of included studies and distribution of radionuclides reported for TAT of SSTR_2_-overexpressing cancers.

The published literature relating to TAT of SSTR_2_-overexpressing cancers was reviewed via keyword database search (Pubmed, Web of Science, Ovid). Results were included up to the date of 1 December 2023. Results were screened and categorised by scope into in vitro, in vivo, clinical, in silico, case report, abstract, or review. Abstracts and reviews were excluded from further analysis. A total of 43 studies were found; the distribution of article scope and radionuclide across the studies is shown in Figure 4.

The majority of the current clinical experience with TAT for PRRT in NETs is with ^225^Ac. Despite theoretical feasibility and applications for other indications, no studies were found relating to ^223^Ra, ^224^Ra or ^227^Th. Most studies used the DOTA chelator (29/43) and the TOC/TATE (37/43)-targeting ligand. Two SSTR antagonists (LM3, JR11) were investigated, in contrast to the conventional agonists [40,60]. Several more recent publications examined SSAs conjugated to a new chelator (via a PEG linker), developed specifically for ^203/212^Pb radiopharmaceuticals, and appear to improve the pharmacokinetic properties and stability of chelation [46,47,49].

### 3.2. Preclinical Studies

An overview of all studies containing in vitro and/or in vivo work is given in Table 2. Analysis of the specific aspects of the studies is continued in the sections below, with a focus on the relationships between activity (MBq), absorbed dose (Gy) and biological endpoint.

#### 3.2.1. In Vitro RBE

Applying RBE to TAT, we may ask what absorbed dose of an α-emitter is required to produce the same biological effect as a low-LET reference radiation such as photons or electrons. Three studies report the RBE of TAT in vitro. A summary of these findings can be found in Table 3, showing the sometimes subtle differences between these studies. Averaging these estimates results in an RBE of 3.9 (SD 1.9) for cell killing with α-particles in vitro.

The RBE can be seen to depend on several parameters, including cell line, reference radiation and biological end point. Dependence on cell line may be due to varying levels of SSTR_2_ expression. The end points considered also differ subtly. Two studies consider the absorbed dose to produce a cell survival of 10% or 20%. Typically, cell survival curves for high-LET radiation such as α-emitters are log-linear functions of absorbed dose, meaning that RBE will vary according to the end point chosen. The parameter RBE2 has been proposed to overcome this shortcoming, and is defined as the ratio of the linear coefficients characterising the high-LET dose-response curve and the low-LET MV photon 2 Gy fraction-equivalent absorbed dose-response curve [79].

Dosimetry in this setting is not routinely carried out and lacks standardization. For example, Chan and collaborators separately consider the absorbed dose due to specific irradiation from the bound radionuclide and non-specific irradiation from the radioactive incubation medium [64]. In the specific case, the MIRD formalism is applied using the MIRDcell software V2.0 [80], in which sub-cellular regions are considered as sources and targets, with dimensions and uptake fractions calculated and used as the basis for dosimetric estimate [81]. For the non-specific scenario, a bespoke Monte Carlo method was applied using the radiation transport code MCNPX. In this way, an estimate of the radiation dose received by the cells (grown as an adherent monolayer) from the radiation distributed throughout the total volume of liquid in the well was made. When estimating RBE, these assumptions should be communicated clearly, and it should be acknowledged that methodological differences may cause discrepancies between estimates [82].

#### 3.2.2. In Vivo Efficacy

A total of 11 studies assessed the efficacy of TAT in vivo, with efficacy being defined according to a range of endpoints. Most commonly (5/11 studies), parameters relating to tumour growth rate were assessed, such as tumour regrowth doubling time and tumour growth delay. Also in 5/11 studies, overall survival (OS) was considered, and 4/11 studies considered tumour size. Other endpoints investigated included cure rate and various potential biomarkers of response (percentage of cells undergoing apoptosis, percentage of *γ*H2AX positive cells, SSTR_2_ expression).

The animal species studied were predominantly mice, apart from one study where Lewis rats were used [72]. It is noted that when assessing efficacy in athymic mice, any response attributable to the immune system cannot be studied. Increasingly, the immune response is known to play an important role in anti-tumour effects [83], leading to a potentially important discordance between the preclinical and clinical settings.

A source of heterogeneity between studies was the tumour model used. Firstly, a range of cell lines was used as a xenograft, most commonly the rat pancreatic cancer cell lines AR42J and CA20948, but also a range of non-small cell lung cancer cells showing SSTR_2_ expression (H69, H727, A549). The fact that these cell lines are being studied preclinically is a sign that the indication for PRRT could soon expand beyond midgut NETs studied in the NETTER-1 trial. Even amongst studies considering the same tumour cell line, the number of cells inoculated and tumour size at the time of PRRT varied significantly, from non-visible to 382 mm^3^ in mice and 1720 mm^3^ in rats. Importantly, none of the cell lines studied represent a model of a human NET. For the best prospect of translation, the level and heterogeneity of SSTR_2_ expression should bear similarity to the clinical scenario.

**Figure 5:**
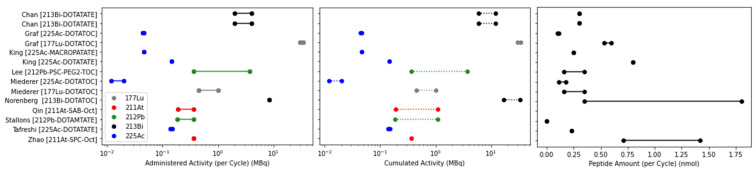
Range of administered activity, cumulated activity and administered peptide mass in preclinical efficacy studies.

Conclusions about the efficacy of TAT, particularly in comparison to β-PRRT, are of limited use if the activities and peptide masses administered are arbitrary. The administered activity should be chosen to maximize the therapeutic index, balancing anti-tumour efficacy against the risk of toxicity. However, few studies selected a therapeutic activity based on a prior activity escalation toxicity study, King [68] and Stallons [74] being the only examples. The majority of studies (8/11) did consider some form of activity escalation when assessing therapeutic response, though a rationale for the activities chosen was rarely given. One study based administered activities on renal dose limits from the literature [46], specifically 27 Gy for β-particles (adjusted according to an RBE of 5 for α-particles), and 11 Gy and 20 Gy based on the results of Chan and collaborators [63]. The range of single cycle activity, cumulated activity and peptide amount *per* cycle are shown in Figure 5. Differences between radiopharmaceuticals are to be expected, given the differing pharmacokinetics and decay scheme energies. However, differences of several orders of magnitude were observed with the same radiopharmaceutical. The administered mass of peptide has been shown to alter biodistribution [49,84], therefore care must be taken in interpreting results if changing peptide mass and radionuclide activity simultaneously. However, Stallons and collaborators found that decreasing the specific activity by a factor of roughly 25 did not significantly alter the tumour uptake in a biodistribution study [74]. Roughly half of the studies (5/11) considered a single administration only. Of the six considering a fractionated regimen, the interval between cycles varied significantly, from a single day (42, 44 [75]) to 14–21 days [72]. Alternating the fractionation regime was shown to have a significant effect on overall survival [46,74].

Despite methodological differences, the results in these studies go some way to explaining the excitement around TAT. A significant anti-tumour response leading to prolonged survival is repeatedly demonstrated across a range of tumours and tumour sizes. In both studies by Chan and collaborators investigating [^213^Bi]Bi-DOTATATE, median overall survival in a treated cohort was extended beyond the length of follow up; in the later study, complete responses were observed [61,63]. Stallons and collaborators and Lee and collaborators demonstrate complete responses to ^212^Pb [74,82], King and collaborators reports complete response following ^225^Ac [68]. However, this effect is not seen evenly across all treated cohorts, stressing the importance of optimising the way these novel radiopharmaceuticals are given.

#### 3.2.3. In Vivo Healthy Tissue Toxicity

While the previous section demonstrates exciting potential efficacy, an acceptable toxicity profile is also a prerequisite for TAT. Firstly, it was observed that whether toxicity was determined in healthy or tumour-bearing animals was a source of discordance between studies. Toxicity was assessed in healthy animals in 3/10 studies, 6/10 in tumour-bearing animals and 1 study considered both scenarios. Given that tumour uptake has been shown to lead to decreased bioavailability in normal tissue [85], this introduces some uncertainty when extrapolating results from healthy animals.

Through appraising the literature, the commonly observed toxicities arising from TAT in the preclinical setting were established. The most common observation was weight loss, a non-specific marker seen in 6/10 studies that assessed toxicity. In each case, weight loss was associated with increasing administered activity.

Evidence of nephrotoxicity, a known adverse effect of PRRT, was also found in 6/10 studies. This was most commonly seen on pathological examination (5/6). Interestingly, 3/10 studies measured changes in blood urea nitrogen (BUN) and creatinine as potential biomarkers of renal injury but found no relationship between these parameters and outcome. This indicates that these commonly used biomarkers are not sensitive markers for nephrotoxicity, meaning more appropriate biomarkers are required, for example neutrophil gelatinase-associated lipocalin (NGAL), as investigated by Chan and collaborators [62] and highlighted as a potential biomarker of tubular damage and long-term nephrotoxicity evaluated by Li et al. [86]. One study did assess renal function via functional imaging with ^99m^Tc-DMSA, but no difference in uptake between treated and control cohorts was observed [61]. Clinically, amino acids are commonly co-administered with PRRT to inhibit reabsorption of the radiopharmaceutical in proximal tubular cells, therefore significantly reducing uptake and radiation-absorbed dose [87]. Of the studies included here, 6/10 did not co-administer amino acids for renal protection. The remaining 4/10 investigated toxicity both with and without amino acids. Chan and collaborators [63] show that the renal-absorbed dose is decreased by a factor of roughly two by the co-administration of L-lysine with [^213^Bi]Bi-DOTATATE, suggesting that the sparing remains relevant in TAT. Conclusions around renal toxicity that do not account for the sparing effect of amino acids should be considered with this potential improvement in mind.

Aside from renal toxicity, haematological toxicity was assessed in 5/10 studies. Stallons and collaborators observed decreased levels of leukocytes, erythrocytes, albumin and bone marrow depletion leading to mortality following the highest activity administration of [^212^Pb]Pb-DOTAMTATE [74]. However, this study also showed that it was possible to manage and overcome this toxicity when using a fractionated administration regimen (3 cycles at 21-day intervals). One study reported mild hypothyroidism as a side effect, shown through low thyroid hormone level in blood sampling, but this was not considered significant. No hepatotoxicity was observed, and no toxicity associated with the administration of unlabelled peptide as a control was reported.

Maximum tolerated activity was investigated in two studies. For [^213^Bi]Bi-DOTATATE, maximum tolerated activity with and without renal protection was determined as 21.7 and 13.0 MBq, given as three or two cycles with one day intervals, respectively. For [^212^Pb]Pb-DOTAMTATE, the maximum tolerated activity was between 0.74 and 1.48 MBq when given as a single administration, roughly an order of magnitude lower than for ^213^Bi. The no-observed-effect level activity was found to be 0.37 MBq, and the highest non-severely toxic dose was 0.74 MBq. Lee et al. did not observe any acute toxicity or lethal effects with activities up to 3.7 MBq using [^212^Pb]Pb-PSC-PEG_2_-TOC, perhaps due to the introduction of the new Pb-specific chelator and PEG_2_ linker and improved chelation and renal clearance of this agent [46]. While not strictly defined as maximum tolerated activity within the study, Miederer and collaborators [69] showed that no histopathologic alterations were found in the kidneys after treatment with [^225^Ac]Ac-DOTATOC at activities below 20 kBq. No studies performed dosimetry to estimate the relationship between absorbed dose and toxicity in a specific organ.

#### 3.2.4. In Vivo Dosimetry

In attempting to understand response and toxicity quantitatively, and potentially relate this knowledge to new contexts, absorbed dose to the tumour and to organs at risk is an important parameter. Clinically, there is a growing body of evidence implying correlation between absorbed dose delivered and therapeutic response [88]. Similarly, renal-absorbed dose is considered a risk factor for long-term renal toxicity after β-PRRT [89]. Five studies estimated absorbed dose to the tumour and six studies estimated absorbed dose to the kidneys in the preclinical setting. The reported absorbed dose coefficients (ADC) are given in Table 4. The absorbed dose coefficient varied depending on the cell line used, even when the tumour size was comparable at the time of administration. The large difference in absorbed dose coefficients for [^225^Ac]Ac-DOTATATE and for [^213^Bi]Bi-DOTATATE reflects the net emission of four α particles *per* decay of ^225^Ac, compared to one *per* decay of ^213^Bi. The ratio of tumour-absorbed dose to kidney-absorbed dose is a useful metric to potentially understand the viability of a perspective therapeutic agent. The highest reported ratio is for [^212^Pb]Pb-PSC-PEG_2_-TOC, where structural modifications to the chelator and linker result in T:K absorbed dose >2.6. Contrastingly, Handula and collaborators conclude that [^225^Ac]Ac-DOTA-JR11 is unsuitable for therapy based on a low T:K of 0.34 [60]. While T:K is a useful parameter, clearly more work is required to properly understand what constitutes effective tumour-absorbed doses and safe renal-absorbed doses for this class of radiopharmaceuticals.

As mentioned, RBE is an important parameter, particularly in comparison with the RBE determined in vitro. This would go some way towards answering the question of whether in vitro radiosensitivity is a relevant parameter in the more complex in vivo setting. Only two studies considered the efficacy of α and *β* radiation head to head, both with [^177^Lu]Lu-DOTATOC as the *β*-emitting radiopharmaceutical. Graf and collaborators showed a growth delay of 20 and 15 days with ^225^Ac and ^177^Lu when tumour-bearing mice were treated with equitoxic activities as determined via MTT assay (44 kBq and 34 MBq, respectively). However, independent in vivo dosimetry was not carried out, meaning RBE could not be estimated [67]. Miederer and collaborators [69] found that treatment with 20 kBq of [^225^Ac]Ac-DOTATOC showed a significantly greater reduction in tumour mass than 1 MBq of [^177^Lu]Lu-DOTATOC, but no in vivo dosimetry was performed to quantify tumour-absorbed dose and estimate RBE directly.

### 3.3. Clinical Applications

An overview of published clinical studies is given in Table 5. Only studies reporting results from a cohort of patients were considered for further analysis, therefore excluding individual case reports.

#### 3.3.1. Clinical Administration Regimen

There is no typical administration regimen for clinical studies of TAT in the published literature. Randomised controlled trials (RCTs) are so far sparse, meaning many patients included in this review were treated according to the local physician’s discretion. Typically a fractionated administration regimen was adopted, except in the study of [^212^Pb]Pb-DOTAMTATE which was initially considered as a single administration before moving to multiple administrations during the study [92]. An overview of the administration regimens used across the included studies is given in Table 6.

#### 3.3.2. Clinical Efficacy

In total, 223 patients were reported across 9 studies. The majority of these patients were treated with [^225^Ac]Ac-DOTATATE (n = 143). Treatment outcome according to RECIST criteria was reported in 6/9 studies, comprising 146/223 patients.

**Figure 6:**
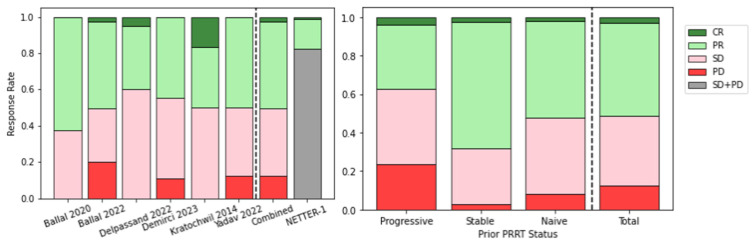
Response following TAT for metastatic NETs (**left**) and stratified by prior PRRT status (**right**) [90,91,92,93,95,97]. CR = complete response, PR = partial response, SD = stable disease, PD = progressive disease.

Combining results from the included studies, the rate of response following TAT was determined and is shown in Figure 6 (left). The objective response rate (ORR), combining complete and partial responses, was 51%. This rate is impressive when compared with the objective tumour response rate reported in the NETTER-1 (complete response 1/101, partial response 17/101) [21].

It was also possible to stratify response rate according to the patient’s disease status prior to PRRT treatment with a β-emitter, also shown in Figure 6 (right). TAT response in patients with stable disease prior to PRRT treatment is better than for those with progressive disease. However, 38% of patients showed an objective response despite being refractory to β-PRRT, demonstrating the potential of TAT to overcome resistance to agents such as [^177^Lu]Lu-DOTATATE.

As well as morphologically, response was observed biochemically via a decrease in chromogranin A (CgA) (secreted by functional NETs), and in quality of life, via improvements in Karnofsky performance status (KPS) [91].

Despite impressive results, it is still unclear as to what the optimal activity for each α-emitting radiopharmaceutical is, and no relationship between tumour-absorbed dose and response has been reported. Imaging is feasible, to varying extents, for each of the α-emitters covered in these studies; therefore, image-based dosimetry should be performed in future patients to better understand how absorbed dose and biological effect are related.

#### 3.3.3. Clinical Toxicity

Experience with β-emitters has shown that the incidence and severity of adverse events from PRRT is modest. However, the toxicity that does occur is often associated with the kidneys, acknowledged as the dose-limiting organ in current clinical practice, and the bone marrow [7]. Incidence of grade IV/V renal toxicity was reported as 9.2% in a series of 1109 patients [99]. Bodei and collaborators [100] demonstrated that in a cohort of 807 patients treated with either [^177^Lu]Lu-DOTATATE, [^90^Y]Y-DOTATOC or a combination of both, renal toxicity was least common in patients receiving ^177^Lu alone. This result may lend itself to speculation that renal toxicity is associated with the range of the β-particle, implying that the lower energy of the ^177^Lu emission is less damaging to potentially radiosensitive structures in the kidney, for example the glomerulus [101]. This would imply that TAT, with the short range associated with the α-particle (approximately 0.1 mm), may prevent renal toxicity in the same manner. However, damage to the tubular cells, via which radiolabelled peptides are re-absorbed, is a known driver of chronic kidney disease [30]. Given this, and the significant difference in physical parameters such as LET, the toxicities associated with TAT cannot be considered identical to the toxicities associated with β-PRRT.

Of the clinical studies included here, toxicity was reported in 7/9 and was most commonly assessed in accordance with the common criteria for adverse events (CTCAE) framework [102]. Toxicity was generally assessed via routine clinical assessment and blood sampling, though one study did perform renal scintigraphy to assess kidney function post-therapy [96]. The length of follow-up varied from 3 to 60 months.

Concerning nephrotoxicity, Delpassand and collaborators [92] reported three serious treatment emergent adverse events (TEAEs) after [^212^Pb]Pb-DOTAMTATE (two in single activity escalation cohorts, deemed unrelated, and one in the multiple cycle cohort in a patient with multiple existing risk factors). Kratchowil and collaborators [95] reported moderate chronic kidney toxicity in patients treated with [^213^Bi]Bi-DOTATOC, evidenced by a 30% decline in glomerular filtration rate (GFR) and a 40% decline in tubular excretion rate (TER) over two years. The same group also reported outcomes in 22 patients treated with [^225^Ac]Ac-DOTATOC with a median follow up of 57 months [96]. Two patients developed terminal kidney failure after >4 years, though both patients presented with prior risk factors. Analysis showed eGFR loss of 8.4 mL/min/year and a TER decrease of 7.6% in the first 6 months after TAT and 14% in the first 18 months. Otherwise, no incidence of renal toxicity was reported, and no study reported a relationship between treatment activity and toxicity.

Low-grade haematological toxicity was reported as the most common treatment-related side effect in two studies by Ballal and collaborators with [^225^Ac]Ac-DOTATATE [90,91], and shown by a statistically significant but recoverable drop in lymphocytes after [^212^Pb]Pb-DOTAMTATE [92]. Treatment with [^225^Ac]Ac-DOTATOC also resulted in activity-dependent thrombocytopenia and leucopenia, and severe grade III/IV toxicity was observed with activities above 44 kBq given as a single cycle. With repeated administrations, no cumulative toxicity effect was observed when given at 4-month intervals, but at 2-month intervals, additive toxicity was observed. The incidence of high-grade haematological toxicity was modest, with seven grade III/IV adverse events reported across the studies.

A wide range of other adverse events was reported, commonly loss of appetite and transient nausea, although these side effects are also associated with the administration of amino acids for renal protection. There was one report of induced Graves’ disease, considered treatment related because thyroid cells may express SSTR_2_ [94].

When attempting to draw conclusions from these studies, it is noted again that these patient cohorts represent a heterogeneous group, many of which have already received β-PRRT. If TAT were to follow prior therapy with a β-emitter, there are many unanswered questions about how this potentially cumulative absorbed dose to healthy organs would affect toxicity and any possible relationship between absorbed dose and response. No studies in this review reported a relationship between administered activity and toxicity, though the incidence of toxicity was low, with the majority of studies reporting no grade III/IV toxicity. This may imply that there is scope for activity escalation, at least in a sub-cohort of patients, to potentially improve the anti-tumour effect. The understanding of the balance between efficacy and toxicity for TAT would be aided by dosimetry, but no studies to date have calculated absorbed doses in clinical α-PRRT. This should be considered a limitation, given that it is known that administration of fixed activities leads to a wide range of absorbed doses to normal organs [103].

A common point made across several studies was that patients with prior risk factors were more likely to suffer treatment-related adverse events, for example renal toxicity. This implies that some level of patient stratification, as implemented in the phase II ILUMINET trial by applying different absorbed dose limits for patients with and without prior risk factors receiving β-PRRT [104], may be beneficial when attempting to widen the therapeutic window for individual cases.

### 3.4. Ongoing Clinical Trials

A phase I trial (NCT03466216) of a [^212^Pb]Pb-DOTAMTATE compound, termed Alphamedix, was sponsored by Radiomedix and completed in 2021. This trial was considered successful and a phase II trial (NCT05153772) of this conjugate is ongoing, with PRRT-naive patients receiving 67.6 µCi/kg *per* cycle. A phase 1/2a dose escalation trial (NCT05636618) of [^212^Pb]Pb-VMT-α-NET, sponsored by Perspective Therapeutics, began enrolment in July 2023, with an expanded indication to include all NETs. One trial using ^225^Ac, sponsored by RayzeBio, is currently recruiting (NCT05477576). In phase 1, the safety, pharmacokinetics and recommended phase 3 dose of RYZ101 ([^225^Ac]Ac-DOTATATE) will be determined in patients who have progressed following ^177^Lu-SSA. Following this, safety and efficacy will be assessed.

## 4. Discussion

Targeted therapy with α-emitters has shown excellent promise in the preclinical setting, and this is beginning to be translated to the clinic. Complete responses to therapy are reported extensively in mouse models, and observed at rates beyond those which can be expected from β-PRRT in the limited clinical experience currently published. Anti-tumour efficacy is demonstrated in patients with progressive disease following prior PRRT with a β-emitter, demonstrating the potential potency of α-emitters for this indication.

However, a review of the relevant literature highlights the lack of understanding as to the relationships between activity, absorbed dose and biological end point at all scales. Better understanding of these relationships can provide the basis for treatment optimisation and individualisation, as well as providing insight into the fundamental radiobiology underpinning the effectiveness of high-LET radiation.

One interpretation of optimisation in the context of any targeted therapy would be to maximise anti-tumour efficacy while maintaining the risk of healthy organ toxicity below an acceptable level. In order to implement this, there must be a fundamental understanding of how tumour response and toxicity are related to the quantity of the specific radiation quality administered. This review shows that these relationships are poorly defined for α-emitters, leading to arbitrary choices of therapeutic activity, comparisons between high- and low-LET radiation at activities that are not equitoxic, and the extrapolation of constraints on absorbed dose from external beam radiotherapy that are unlikely to translate to TAT [105].

No clinical estimates of absorbed doses to the tumour or to organs at risk in humans have been published. Dosimetry was performed in a minority of cases in both the in vitro and in vivo settings. Even when absorbed doses were estimated, methodologies differed substantially, potentially reducing the scope for comparison between studies. Uncertainties associated with estimates of absorbed dose were also rarely included, despite their importance for interpretation of the result [106]. It should also be acknowledged that the most common dosimetry methods in the preclinical studies reviewed here are macroscopic and aim at characterising mean organ-absorbed dose. Given the likely heterogeneous distribution of the radiopharmaceutical on the cellular scale, it may be that mean organ-absorbed dose is too crude a measure to correlate with biological endpoint, and alternative approaches including organ sub-unit dosimetry and microdosimetry are necessary [107,108]. Clearly, standardised methods for dosimetry and uncertainty calculation should be included in future work.

Impressive preclinical responses indicate that TAT has a high potential for anti-tumour efficacy in SSTR_2_-overexpressing cancers. However, clinical imaging studies have demonstrated that uniformly positive receptor expression may not be typical in NET patients [109]. Given the short range of the α-particle, cells with lower receptor expression than neighbour high-expressing cells are less likely to be irradiated due to the crossfire effect. This demonstrates the role of imaging in identifying potentially sub-optimal candidates for TAT, and the potential role of combination therapies in future patients in a so-called ‘cocktail approach’ [110].

## 5. Conclusions

In this review, we hope to have highlighted the substantial benefit that TAT could offer patients for whom PRRT is indicated. We have laid out the radiobiological advantages of α-particles over β-particles due to their high-LET and draw attention to impressive results with a moderate toxicity profile in both the preclinical and clinical settings. The ORR of TAT in a population of mixed prior PRRT status was 51%, and efficacy is demonstrated in patients who are refractory to β-PRRT. However, we also demonstrate how research in this area is discordant and treatment remains non-optimised. It is hoped that theragnostic imaging, dosimetry and a better understanding of the relationships between absorbed dose, therapeutic response and toxicity will facilitate this optimisation to provide benefit for future patients.

## Figures and Tables

**Table 1:** Relevant radionuclides for TAT, including in vivo alpha generators. Blue indicates isotopes with potential for clinical translation, green indicates stable isotopes. Emissions listed are not exhaustive but represent most of the energy in the decay scheme. ε = electron capture. Energy of β-particles refers to the mean energy. Decay data from ENSDF database as of February 2024 (http://www.nndc.bnl.gov/ensarchivals, accessed on 22 February 2024).

Parent	Daughters	T_1/2_	Decay Type	Energy (MeV)	Yield	Imaging
^227^Th		18.7 d	α	5.76, 5.98, 6.04	0.20, 0.23, 0.24	γ: 236 keV (0.13)
	^223^Ra	11.4 d	α	5.60, 5.72	0.25, 0.51	γ: 269 keV (0.13)
	^219^Rn	3.96 s	α	6.55, 6.82	0.13, 0.79	γ: 271 keV (0.11)
	^215^Po	1.78 ms	α	7.39	1.00	-
	^211^Pb	36.1 min	β-	0.16, 0.47	0.06. 0.91	γ: 405 keV (0.04)
	^211^Bi	2.14 min	α	6.28, 6.62	0.16, 0.84	γ: 351 keV (0.13)
	^207^Tl	4.77 min	β-	0.493	1.00	-
	^207^Pb	Stable				
^225^Ac		10.0 d	α	5.79, 5.83	0.18, 0.51	-
	^221^Fr	4.80 min	α	6.13, 6.24 6.34	0.15, 0.01, 0.83	γ: 218 keV (0.13)
	^217^At	32.6 ms	α	7.07	1.00	-
	^213^Bi	45.6 min	α (0.02)	5.86	0.02	γ: 440 keV (0.26)
			β- (0.98)	0.32, 0.49	0.30, 0.67	
	^213^Po	3.72 µs	α	8.38	1.00	-
	^209^Tl	2.16 min	β-	0.660	0.97	γ: 117 keV (0.76)
	^209^Pb	3.23 h	β-	0.198	1.00	-
	^209^Bi	2.0 × 10^19^ y	α	2.88, 3.08	0.01, 0.99	-
	^205^Tl	Stable				
^224^Ra		3.66 d	α	5.45, 5.69	0.05, 0.95	γ: 241 keV (0.04)
	^220^Rn	55.6 s	α	6.29	0.99	-
	^216^Po	144 ms	α	6.78	1.00	-
	^212^Pb	10.6 h	β-	0.41, 0.93, 0.17	0.05, 0.81, 0.14	-
	^212^Bi	60.6 min	α (0.36)	6.05, 6.09	0.25, 0.10	γ: 727 keV (0.07)
			β- (0.64)	0.53, 0.83	0.04, 0.55	
	^212^Po	17.1 ns	α	10.2	0.42	-
	^208^Tl	3.05 min	β-	0.44, 0.54, 0.65	0.24, 0.22, 0.49	γ: 277 keV (0.07)
	^208^Pb	Stable				
^211^At		7.21 h	α (0.42)	5.87	0.42	X: 77–92 keV
			ε (0.58)	-	-	-
	^211^Po	0.52 s	α	7.45	0.99	-
	^207^Bi	31.6 y	ε	-	-	γ: 570 keV (0.98)
	^207^Pb	Stable				
^149^Tb		4.12 h	α (0.17)	3.97	0.17	β+: 639 keV (0.04)
			ε (0.83)			γ: 165 keV (0.27)
	^149^Gd	9.28 d	ε	-	-	γ: 150 keV (0.48)
	^149^Eu	93.1 d	ε	-	-	-
	^149^Sm	Stable				
	^145^Eu	5.93 d	ε	-	-	β+: 740 keV (0.02)
	^145^Sm	340 d	ε	-	-	-
	^145^Pm	17.7 y	α (2.8 × 10^−7^)	2.24	2.80 × 10^−7^	-
			ε (1.00)	-	-	-
	^145^Nd	Stable				
	^141^Pr	Stable				

**Table 2:** Overview of in vitro and in vivo studies in α-PRRT. OS = overall survival, D_10_ = absorbed dose to reduce survival to 10%, MPC = murine pheochromocytoma cell.

Author	Radiopharmaceutical	Aim	Findings
Chan [61]	[^213^Bi]Bi-DOTA-TATE	Determine whether TAT efficacy in vivo is related to tumour size in two SSTR_2_ +ve cell lines.	Improved OS, increased tumour doubling time vs. control in small (50 mm^3^) and large (200 mm^3^) CA20948 and H69 tumours. Several cures in small tumour cohort. No toxicity.
Chan [62]	[^213^Bi]Bi-DOTA-TATE	Investigate optimal radiolabelling conditions (peptide amount, quencher, pH) for [^213^Bi]Bi-DOTATATE.	>3.5 nmol DOTATATE required for >99% incorporation with 100 MBq ^213^Bi. Optimised conditions: pH = 8.3, TRIS = 0.15 mol/L in 800 µL. Ascorbic acid (0.9 mmol/L) required to avoid radiolysis.
Chan [63]	[^213^Bi]Bi-DOTA-TATE	Evaluate the therapeutic effect of TAT with and without renal protection using L lysine in vivo.	MTA in healthy mice = 13, 21.7 MBq with/without renal protection. In tumour-bearing, median OS > 30 d at 17 MBq, severe weight loss and mortality at 33 MBq. Renal protection improved OS.
Chan [64]	[^213^Bi]Bi-DOTA-TATE	Develop methods to determine relationship between absorbed dose and cell killing in vitro. Compare cytotoxicity across radiations in various cell lines.	In CA20948, D_10_ = 3 Gy, 18 Gy and 5 _Gy_ for [^213^Bi]Bi-DOTATATE, [^177^Lu]Lu-DOTATATE and ^137^Cs. In BON, [^177^Lu]Lu-DOTATATE had no effect, D_10_ for [^213^Bi]Bi-DOTATATE, ^137^Cs = 2.5 Gy, 4.5 Gy.
Chapeau [65]	[^212^Pb]Pb-eSOMA-01	Develop new octreotate derivatives with non-DOTA chelators and assess their potential for TAT of NETs with Pb.	New SSTR_2_-targetting ligands labelled successfully with ^212/203^Pb, eSOMA-01 showed favourable biodistribution compared to DOTAM-TATE.
Cieslik [66]	[^225^Ac]Ac-L1-TATE	Assess feasibility of L1 as chelator with ^177^Lu, ^211^At, ^225^Ac in two SSTR_2_ +ve cell lines, evaluate biodistribution in MPC tumour bearing mice.	L1 can bind radionuclides for imaging and therapy. Preferable fast and mild labelling compared to DOTA. [^225^Ac]Ac-L1 produced with molar activity > 0.25 MBq/nmol.
Graf [67]	[^225^Ac]Ac-DOTA-TOC	Assess *γ*H2AX foci formation as biomarker of cytotoxicity and response to [^225^Ac]Ac-DOTATOC and [^177^Lu]Lu-DOTATOC in vitro and in vivo.	High tumour control rate with single treatment of both agents. Number of γH2AX foci correlated with apoptosis (in vitro) and tumour growth, showing potential as biomarker.
Handula [60]	[^225^Ac]Ac-DOTA-JR11	Investigate potential of [^225^Ac]Ac-DOTA-JR11 (antagonist) for therapy of NETs via mouse model.	Low tumour-to-kidney ratio of absorbed dose is limiting for therapeutic use of [^225^Ac]Ac-DOTA-JR11.
King [68]	[^225^Ac]Ac-MACROPA-TATE	Synthesise and characterise MACROPA TATE, compare performance with DOTA TATE in labelling efficiency, stability, binding, efficacy.	[^225^Ac]Ac-MACROPATATE showed higher renal and liver uptake and toxicity at lower activities, DOTATATE deemed superior.
Lee [46]	[^212^Pb]Pb-PSC-PEG_2_-TOC	Improve SSTR_2_ targeting over DOTA-based conjugates via click-chemistry-based cyclization, improved chelator design and insertion of PEG linkers.	Development of lead-specific chelator (PSC) and insertion of PEG linkers results in improved tumour uptake, retention and quicker renal clearance, and dose-dependent therapeutic effect with acceptable toxicity.
Li [47]	[^212^Pb]Pb-PSC-PEG-TOC	Characterise Pb-specific chelator for radiolabelling yield, stability and in vivo biodistribution.	^212^Pb and ^212^Bi stably incorporated in PSC-PEG-TOC. Biodistribution of ^212^Pb/^212^Bi-PSC-PEG-TOC were comparable. ^203/212^Pb showed comparable biodistribution.
Miederer [69]	[^225^Ac]Ac-DOTA-TOC	Compare biodistribution, toxicity and anti-tumour effect of [^225^Ac]Ac-DOTATOC and [^177^Lu]Lu-DOTATOC.	Activities > 30 kBq of ^225^Ac-induced tubular necrosis, weight loss. ^225^Ac (20 kBq) showed improved tumour growth delay vs. ^177^Lu (0.45 MBq).
Müller [70]	[^149^Tb]Tb-DOTA-NOC	Letter to the editor to highlight the potential of ^149^Tb for ‘α PET’.	High quality PET image of mouse injected with 7 MBq [^149^Tb]Tb-DOTANOC showing high tumour uptake.
Nayak [71]	[^213^Bi]Bi-DOTA-TOC	Compare binding, cytotoxicity, induction of apoptosis between [^213^Bi/^177^Lu]Lu-DOTATOC in human pancreatic adenocarcinoma cells.	RBE of [^213^Bi]Bi-DOTATOC, [^177^Lu]Lu-DOTATOC relative to ^137^Cs = 3.4, 1.0. ^213^Bi induced greater release of apoptosis markers in Capan-2 cells.
Norenberg [72]	[^213^Bi]Bi-DOTA-TOC	Evaluate quantitative labelling methods, stability, biodistribution, safety, and efficacy in vivo.	Activity-related decrease in tumour growth rate observed (>11 MBq). Mild acute but no chronic nephrotoxicity. No haemato-toxicity.
Pretze [49]	[^212^Pb]Pb-PSC-PEG_2_-TOC	Investigate the influence of different molar activities of [^203/212^Pb]Pb-PSC_2_-TOC on cell uptake.	Uptake increased with molar activity, 15–40 MBq/nmol showed highest cell uptake.
Qin [73]	[^211^At]At-SAB-Oct	Develop octreotide SAB conjugate to be labelled with ^211^At and evaluate therapeutic efficacy against SCLC.	Anti-tumour response against SCLC model demonstrated, with acceptable toxicity profile.
Stallons [74]	[^212^Pb]Pb-DOTAM-TATE	Determine binding and cell kill in vitro. Assess biodistribution in vivo. Establish tolerable regimen and efficacy as mono and combination therapy.	Non-toxic at <45 µCi, toxicity overcome by fractionation into 3 cycles. 79% cure rate with 3 × 10 µCi in combination with 5FU. Benefits of ascorbic acid and nephro protection demonstrated.
Tafreshi [75]	[^225^Ac]Ac-DOTA-TATE	Assess toxicity, biodistribution, dosimetry and efficacy in lung neuroendocrine model (H727/H69) in vivo.	Chronic progressive nephropathy at >111 kBq. Single admin produced tumour growth delay and reduction in tumour volume vs. control.
Vaidyanathan [76]	[^211^At]At-GIMBO	Synthesise octreotate analogue with guanidine-containing template for ^211^At labelling, assess in comparison with Glu-TOCA in vitro and in vivo.	Single step process to synthesise radioiodinated and astatinated octreotide analogue with positive template reported. Affinity for SSTR_2_ demonstrated, but high uptake in normal tissue is limiting.
Wharton [77]	[^225^Ac]Ac-H_4_noneupaX-TATE	Develop novel bifunctional chelator capable of complexing ^225^Ac and ^155^Tb for theragnostics.	H_4_noneupaX was characterised, then labelling of ^225^Ac and ^155^Tb assessed. SPECT/CT imaging of ^155^Tb demonstrates potential as theragnostic pair isotope for ^225^Ac therapy.
Zhao [78]	[^211^At]At-SPC-TOC	Investigate possible use of ^211^Ac-labelled octreotide to treat NSCLC.	[^211^At]At-SPC-octreotide showed elevated and activity-dependent apoptosis induction compared to PBS, cold peptide and unlabelled ^211^At.

**Table 3:** Overview of in vitro RBE studies for α-emitters. D_10,20_ = absorbed dose required for 10, 20% cell survival. ED_50_ = activity concentration required for 50% cell survival. ^177^Lu is included to demonstrate reported equivalence to irradiation with ^137^Cs.

Author	Cell Line	Radiopharmaceutical	Reference Radiation	End Point	RBE
Chan [64]	CA20948 (rat pancreatic)	[^213^Bi]Bi-DTPA	^137^Cs	D_10_	2.0
		[^213^Bi]Bi-DOTATATE	^137^Cs	D_10_	1.5
		[^213^Bi]Bi-DOTATATE	[^177^Lu]Lu-DOTATATE	D_10_	5.4
		[^213^Bi]Bi-DOTATATE	[^177^Lu]Lu-DOTATATE	D_10_	5.7
	BON (human carcinoid)	[^213^Bi]Bi-DTPA	^137^Cs	D_10_	1.8
		[^213^Bi]Bi-DOTATATE	^137^Cs	D_10_	1.7
Graf [67]	AR42J (rat pancreatic)	[^225^Ac]Ac-DOTATOC	[^177^Lu]Lu-DOTATOC	ED_50_ (kBq/mL)	5.5
Nayak [71]	Capan-2 (human pancreatic)	[^213^Bi]Bi-DOTATOC	^137^Cs	D_20_	3.4
		[^177^Lu]Lu-DOTATOC	^137^Cs	D_20_	1.0

**Table 4:** Summary of preclinical tumour- and kidney-absorbed dose coefficients for TAT. ADC = absorbed dose coefficient. Nephroprotection given as administration of L-lysine before TAT. T:K = tumour-to-kidney ratio.

Author	Radiopharmaceutical	Tumour Bearing	Cell Line	Nephro-Protection	ADC (Gy/MBq)		T:K
					Tumour	Kidneys	
Chan [61]	[^213^Bi]Bi-DOTATATE	+	CA20948	−	0.8	1.6	0.49
		+	H69	−	0.5	2.0	0.23
Chan [63]	[^213^Bi]Bi-DOTATATE	+	AR42J	+	0.7	0.6	1.18
		+	AR42J	−	0.7	1.1	0.64
		−	N/A	+	N/A	0.5	N/A
		−	N/A	−	N/A	1.0	N/A
Chapeau [65]	[^212^Pb]Pb-DOTAM-TATE	+	H69	−	26.6	140.0	0.19
	[^212^Pb]Pb-eSOMA-01	+	H69	−	35.5	121.7	0.29
	[^212^Pb]Pb-eSOMA-02	+	H69	−	14.7	147.4	0.10
Handula [60]	[^225^Ac]Ac-DOTA-JR11	+	H69	−	328.5	952.6	0.34
Lee [46]	[^212^Pb]Pb-DOTA-TOC	+	AR42J	+	2.4	7.0	0.35
	[^212^Pb]Pb-PSC-TOC	+	AR42J	+	9.2	5.4	1.70
	[^212^Pb]Pb-PSC-PEG_2_-TOC	+	AR42J	+	12.7	6.2	2.04
	[^212^Pb]Pb-PSC-PEG_2_-TOC	+	AR42J	+	8.7	3.2	2.69
Tafreshi [75]	[^225^Ac]Ac-DOTATATE	−	N/A	−	N/A	6.8	N/A

**Table 5:** Overview of clinical studies in targeted alpha therapy. QoL = quality of life, PR = partial response, SD = stable disease, PD = progressive disease, TEAE = treatment emerging adverse event, CE-US = contrast enhanced ultrasound, CE-CT = contrast enhanced CT. * n = 14 total, n = 3 ^213^Bi.

Author	Indication	Radiopharmaceutical	N	Aim	Findings
Ballal [90]	GEP-NETs	[^225^Ac]Ac-DOTA-TATE	32	Present early results on safety, efficacy, QoL following TAT in patients stable or refractory to [^177^Lu]Lu-DOTATATE	Morphological response assessed in 24/34 patients, n = 15 PR, n = 9 SD. No disease progression. Therapy was well tolerated in this population.
Ballal [91]	GEP-NETs	[^225^Ac]Ac-DOTA-TATE	91	Evaluate long-term outcome of TAT in GEP-NET patients in mixed population of PRRT naive and pre-treated.	TAT improved OS, even in patients refractory to prior ^177^Lu, with transient and acceptable toxicity.
Delpassand [92]	GEP-NETs	[^212^Pb]Pb-DOTAM-TATE	20	Establish safety of ^212^Pb-DOTAM-TATE in phase 1 dose-escalation study.	TAT well tolerated, no serious TEAEs related to the study drug. ORR of 80% at 2.50 MBq/kg/cycle, showing potential benefit over approved therapies.
Demirci [93]	NETs	[^225^Ac]Ac-DOTA-TATE	11	Retrospective study including 11 patients with NETs of different primary sites treated with [^225^Ac]Ac-DOTA-TATE.	Nine patients had PET/CT follow up. No grade III/IV toxicity, 4/9 partial response, 8/9 disease control. ^225^Ac is safe and effective in treatment of patients refractory to β-PRRT.
Giesel [94]	Hepatic NET mets	[^213^Bi]Bi-DOTA-TOC	14 *	Investigate the role of contrast enhanced ultrasound in monitoring tumour response to α/β PRRT.	CE-US comparable to CE-CT and suitable for monitoring PRRT response. Decrease in perfusion indicative of tumour response.
Kratochwil [95]	NETs	[^213^Bi]Bi-DOTA-TOC	8	Report first in-human experience in PRRT pre-treated patients with [^213^Bi]Bi-DOTA-TOC.	Specific tumour uptake shown on imaging. TAT produced enduring response with moderate nephrotoxicity, is effective against β-refractory disease.
Kratochwil [96]	NETs	[^225^Ac]Ac-DOTA-TOC	39	Estimate optimal single cycle and cumulative activity for [^225^Ac]Ac-DOTA-TOC.	~20 MBq/cycle (4-month interval) and cumulative activity ≤ 60–80 MBq avoided acute and chronic grade III/IV haemato-toxicity, some chronic renal toxicity.
Yadav [97]	Metastatic paraganglioma	[^225^Ac]Ac-DOTA-TATE	9	Evaluate the efficacy and safety of TAT in advanced stage paragangliomas.	50% PR, 37.5% SD, 12.5% PD, with symptoms decreased. No grade III/IV renal or haematological toxicity. Benefit even in patients refractory to β-PRRT.
Zhang [98]	NETs	[^225^Ac]Ac-DOTA-TOC	10	Discuss experience with first-in-human use of novel radiopharmaceuticals, including [^225^Ac]Ac-DOTA-TOC, at Bad Berka.	α-PRRT was well tolerated and effective, including in one patient treated intra-arterially.

**Table 6:** Summary of clinical administration regimen. Med = median. Amino acids = lysine, arginine. Diuretic = hydrochlorothiazide. Radiosensitiser = capecitabine.

Author	Radiopharmaceutical	Activity/Cycle (MBq)	N Cycles	Interval (Weeks)	Cumulative Activity (MBq)	Co-Admin
Ballal [90]	[^225^Ac]Ac-DOTA-TATE	0.1/kg (8/80 kg)	1–4	8	23 (8–33)	Amino acid
Ballal [91]	[^225^Ac]Ac-DOTA-TATE	0.1/kg (8/80 kg)	1–10 (med = 4)	8	36 (22–59)	Amino acid, radiosensitiser
Delpassand [92]	[^212^Pb]Pb-DOTAM-TATE	1.13/kg (90/80 kg)	1	8	84	Amino acid
		1.48/kg (118/80 kg)	1	8	112	Amino acid
		1.92/kg (154/80 kg)	3	8	406	Amino acid
		2.50/kg (200/80 kg)	4	8	791	Amino acid
Demirci [93]	[^225^Ac]Ac-DOTA-TATE	0.1–0.12/kg (8–9.6/80 kg)	1–3	18	N/A	Amino acid
Giesel [94]	[^213^Bi]Bi-DOTA-TOC	N/A	N/A	N/A	N/A	N/A
Kratochwil [95]	[^213^Bi]Bi-DOTA-TOC	1000–10,500	1–5 (med = 4.5)	8	45	Amino acid
Kratochwil [96]	[^225^Ac]Ac-DOTA-TOC	6–60	1–5 (med = 4.5)	8–52 (med = 16)	15,800 (3300–20,600)	Amino acid, diuretic
Yadav [97]	[^225^Ac]Ac-DOTA-TATE	0.1/kg (8/80 kg)	2–9 (med = 3)	8	42.4 (15.5–86.6)	Amino acid, radiosensitiser
Zhang [98]	[^225^Ac]Ac-DOTA-TOC	N/A	N/A	N/A	N/A	N/A

## Data Availability

Authors would be happy to share data upon request.
